# Locomotor Ataxy

**Published:** 1888-12

**Authors:** Clarence Willard Butler

**Affiliations:** Montclair, N. J.


### LOCOMOTOR ATAXY.—A CLINICAL CASE.

Clarence Willard Butler, M. D., Montclair, N. J.

Mr. A. C. B———, the patient, is fifty-seven years of age, dark complexion, medium height, and of rather full habit, but without excessive adipose. He retired from an active and successful business life fourteen or fifteen years ago. For the last few years he has had a good deal of care and anxiety over financial matters, from all of which he has recently been entirely relieved, however. His father suffered from gout; but aside from this the meagre family history which he is enabled to furnish is exceptionally good. Two of his sons have developed epileptiform convulsions, reflex from peripheral irritation. (Hæmorrhoids in one, jealousy and probably masturbation in another.) Both are well now, however; cured by the homœopathic remedy.

Mr. B——— has always been a very healthy man. His habits are and have always been correct. Has never had venereal disease; is abstemious as to the use of alcoholics, and moderate in the use of tobacco (smoking).

In 1868 a railway car in which he sat was precipitated, with a faulty bridge, into a ravine, and he suffered some severe cuts about the head and face, and was much bruised, but broke no bones, and was not conscious of any spinal injury, nor do I think he suffered any.

He has had no severe illnesses. Twice during the last two years I have treated him through attacks of gout, but neither was severe, and both yielded promptly to medication. For the last four years he has been troubled with frequent calls to urinate. This condition is worse at night, is wholly painless, and examinations of his urine have given no signs of kidney disease. Medication has relieved, but not cured him. The beginning of his present trouble he refers to September of 1887. At that time, on attempting to rise one morning, he found himself very dizzy; he succeeded in dressing, however, and ate a good breakfast. Shortly after eating it he vomited suddenly, without nausea and without effort. His vertigo pursued him throughout the day, but under *Conium* it disappeared. Once or twice afterward, within the next two months, he had similar attacks, but less severe. About this time he noticed that he became tired very easily while taking his usual exercise (walking), and in his frequent visits to the bath-room at night he was uncertain in his movements in the darkness, and was obliged to make a light in order to go safely. In spite of these symptoms, however, he did not consult a physician until the middle of December. When called to see him at that time, the evidences of spinal disease were pronounced, and in spite of my best care he grew very rapidly worse until February 5th, 1888, when the following grave conditions presented themselves:

Inability to walk from loss of co-ordinating power over his legs; the ataxic gait, which was plainly noticeable in December, having increased to this extent. The patellar and ankle tendon reflexes are entirely lost. On attempting to stand with his eyes closed he sways, and falls at once unless supported. When his legs are placed in different positions he is unable to tell where they are unless he can see them, and any endeavor to place either leg in a required position is awkwardly and imperfectly made, and is usually unsuccessful, while all involuntary movements of the legs present the exaggerated impulse and imperfect control characteristic of his malady. My notes of the case mention areas of cutaneous anæsthesia, but, unfortunately, do not locate them. The upper extremities are unaffected. At no time has he suffered from any visual complication, and to me an interesting and unusual feature of the case is the entire absence of peræsthesiac manifestations referable to the soles of the feet, sensation in these having been normal throughout. No soreness or tenderness of any portion of the spine. Inquiry respecting his sexual appetite shows that he has had but little passion for a number of years, and has considered himself "out by age."

He presents a perfect history of the "lightning-like pains" which have pursued him from time to time irregularly for two or three years. He has supposed them to be rheumatic in their character, and dosed himself domestically for them. His pulse is one hundred, regular, full, and quick. Temperature normal.

His bowels are obstinately constipated, though the stool when evacuated does not present any unnatural appearance. The urinary trouble which I have already mentioned, frequency of micturition, has largely, but not entirely, disappeared. Those symptoms which trouble him most are great weakness and debility, and this weakness is especially severe in the legs, particularly in the calves of the legs, where it is associated with a sensation of *stiffness* and soreness. The sensations in the calves of the legs are the most frequently complained of, and seem the most annoying to the patient of all his discomforts. He is much annoyed by a sensation as of a belt tightly drawn around the abdomen just above umbilicus; the same sensation is felt at times about the chest below the nipple line. This latter is not constant, however, and never severe, while the former is always present and often painfully severe.

Insomnia. On retiring at nine or half-past nine P. M. he goes to sleep and sleeps until half-past eleven or twelve o'clock, when he awakens usually with a heavy, dull, hard pain low in the abdomen. This continues until toward daylight, when it gradually wears away. It is accompanied with much flatulence, with noisy eructations and dejections of tasteless and odorless gas, the escape of which affords temporary relief to his pains. These pains do not appear in the day-time. During the day he is sleepy, and succeeds in getting some rest (sleep), but the whole amount of his sleep will not average more than five hours out of the twenty-four. He presents, in addition to these especially troublesome symptoms, many others. Mentally he evidences no loss of strength. He is taciturn, almost apathetic, and at the same time, in spite of himself, his thoughts run upon committing suicide as "the easiest way out" of his troubles. His appetite is lost and he has no taste; is not especially thirsty. His tongue is red, *dry*, and divided by numerous little cracks into small, irregular squares. He has little or no headache, but suffers at times with a sense of confusion. During sleep his face is puffed and red, and his breathing deep and noisy. All his symptoms are worse at night.

Many sensations of formication, numbness, etc., are experienced in his legs, principally in the calves and thighs. During the past six weeks he has taken at various times Anac., Sulph., Gelsem., and Hyos., all without benefit, though they were seemingly indicated. On this day, satisfied that Anac., his last medicament, was doing him no good, I made my examination for a new prescription, and elicited the symptoms already enumerated. The mental condition, the noisy evacuations of gas with relief of his symptoms therefrom, the extreme weakness, and *sensation* of great debility, called my attention to Argent. nit. A further study of this drug reveals the following similar symptoms:

Taciturnity; apathy; suicidal tendency.

Staggering gait in the dark.

Dull hearing, with ringing in the ears.

Great debility of the legs.

Dry tongue.

Anorexia—taste lost.

*Noisy ejections of tasteless and odorless gases,* with relief of his symptoms therefrom.

Frequent urination

Sensation of constriction of chest and abdomen, as if tightly bound.

Impotence (?).

Stiffness (rigidity) of calves.

*Great weakness and debility of calves.*

Great general weakness and debility.

Sleeplessness; drowsy during the day.

Many dreams, with restless sleep.

Sensation of heaviness in the abdomen.

Many symptoms of weakness of the legs.

Regarding Argent. nit. as the most similar remedy from this study of it in relation to the case, I gave one dose of it in a high potency (CM.—H. S. Johnstone) dry on my patient's tongue and Sac. lac. *ad lib.*

February 6th.—No change.

February 7th.—Possibly a little more sleep and less abdominal pain; otherwise no change

February 8th.—Undoubted improvement in respect of all his sufferings. He slept until half-past-one A. M., again about two hours, and his abdominal pains were less. Has slept nearly all the morning. Bowels moved without aid. He feels encouraged; is sure the medicine is helping him. A detail of the quotidian history of this case for the next three months would be tedious and profitless. From the 8th day of February, his improvement was steady and uninterrupted until March 16th. On that date he presented the following condition:

Mentally cheerful and hopeful. Suicidal thoughts all gone. He walks all over the house, going down-stairs twice daily to his meals. The gait is much better though still presenting the peculiar ataxic character in some degree. Tendon reflexes unchanged. His sense of debility has largely disappeared. He tires easily, but sleeps well, all the nightly pains having disappeared. The sensation of constriction about the abdomen is still present but less troublesome. The old symptom of frequent urination has returned and is very annoying; also he has had some of the rheumatic pains—the "fulgurant pains"—in his legs on one or two occasions. It is noticeable that the sleeplessness, the severe abdominal pains, and the noisy evacuation of gas, which were among the last symptoms to appear before the February prescription, were those which disappeared first and most completely after that prescription.

Improvement not having ceased, and especially as most gratifying indications of the favorable action of the remedy were present, viz., the disappearance of symptoms in inverse order of their appearance, and the return of symptoms which had once been present in the case and which had disappeared as the patient grew worse, I did not change the remedy nor did I repeat the dose. On the 7th day of April, a careful examination of my patient's symptoms in detail showed that improvement had ceased and certain of his symptoms had grown worse, notably the sense of constriction about the abdomen. I deemed it therefore wise to repeat the remedy, no other being indicated, and I gave it in the same potency in water, a spoonful hourly until twelve doses were taken and then stopped. Improvement manifested itself again within twenty-four hours of this time and continued uninterruptedly until May 12th, when an examination revealed the fact that Mr. B——— could now stand and walk with his eyes closed; that the patellar reflexes had returned (ankle not examined); that no sensations of pain, no symptoms of his former disease were left except some muscular weakness. He considered himself well, and I discharged him from my further care.

This case I have presented because I regard it as interesting and instructive for several reasons.

Etiologically because it adds another to the not too many instances where the gouty diathesis may be considered as a factor. Again, the appearance of the neuropathic tendency, not otherwise traceable in the family history, in the patient's children. Semeiologically, I have already spoken of the absence of perverted sensibility of the soles of the feet. My experience with the disease is not large, but I have never met with another case where normal sensation was retained in this locality.

The rapid course of the disease was also worthy of comment. In six weeks, from a condition of difficulty of locomotion but still of ability to walk unaided about the house and upon the sidewalk for distances of an eighth or a quarter of a mile, so markedly had the disease progressed that he was scarcely able to stand even while watching himself closely, and was wholly unable to take a step without the aid of an attendant.

And lastly, because it presents a case of serious and usually incurable disease promptly, rapidly, and favorably affected by the homœopathic remedy.

It may be urged that sufficient time has not yet elapsed to make his permanent recovery a certainty. I can only say that within three weeks I met him on the street more than a mile from his residence, walking briskly with no trace of his former ataxic awkwardness, and received his assurance that he felt perfectly well. The disappearance of the more marked diagnostic signs of the disease has been already mentioned. Whether well or not, there can be no doubt that the Argent. was the cause of his improved condition, because:

1. His improvement commenced shortly after its administration, and, when improvement subsequently ceased, it was renewed at once by the repetition of this drug.

2. Because the symptoms disappeared in inverse order of their appearance.

3. Because of the reappearance, as improvement was established, of symptoms which had once been present, which had disappeared during the graver aspects of the case. The last two methods of proof of favorable drug-action have been confirmed by many physicians since Hahnemann first called attention to them, are most convincing to the observing homœopath, and will need no elaboration in a gathering of homœopathic physicians. —*Clinical Bureau American Institute*, 1888.

